# HiTEC: a connectionist model of the interaction between perception and action planning

**DOI:** 10.1007/s00426-016-0803-0

**Published:** 2016-09-12

**Authors:** Pascal Haazebroek, Antonino Raffone, Bernhard Hommel

**Affiliations:** 1 Institute of Psychology, Leiden University, Wassenaarseweg 52, 2333 AK Leiden, The Netherlands; 2 Department of Psychology, Sapienza University of Rome, Rome, Italy

## Abstract

Increasing evidence suggests that perception and action planning do not represent separable stages of a unidirectional processing sequence, but rather emerging properties of highly interactive processes. To capture these characteristics of the human cognitive system, we have developed a connectionist model of the interaction between perception and action planning: HiTEC, based on the Theory of Event Coding (Hommel et al. in Behav Brain Sci 24:849–937, 2001). The model is characterized by representations at multiple levels and by shared representations and processes. It complements available models of stimulus–response translation by providing a rationale for (1) how situation-specific meanings of motor actions emerge, (2) how and why some aspects of stimulus–response translation occur automatically and (3) how task demands modulate sensorimotor processing. The model is demonstrated to provide a unitary account and simulation of a number of key findings with multiple experimental paradigms on the interaction between perception and action such as the Simon effect, its inversion (Hommel in Psychol Res 55:270–279, 1993), and action–effect learning.

## Introduction

Coordinating our actions in response to environmental demands is an important cognitive activity. Indeed, actions that are not guided by perception would not only be inefficient but might also be rather dangerous. In general, natural environments offer an overwhelming number of perceivable objects, and natural bodies allow for a virtually unlimited number of different responses. Intriguingly, our cognitive system usually seems to cope quite well with this complexity. It is generally hypothesized that the task context triggers the implementation of a task set (Monsell, 1996) that focuses the cognitive system on relevant perceptual events and appropriate actions. It is, however, unclear how such a task set may configure the cognitive system—in terms of representations and processes—to effectively coordinate our actions in response to stimuli.

Traditionally, responding to stimuli in our environment has theoretically been conceived as a sequence of separable stages of processing (e.g., Donders, 1868; Neisser, 1967; Sternberg, 1969), such as ‘perceptual analysis’, ‘decision making’, ‘response selection’, and ‘response execution’ (Ward, 2002). Interestingly, empirical findings in psychology have demonstrated that parts of human information processing do not seem to involve conscious cognitive decision making. Features of perceived objects (such as location, orientation, and size) can influence actions *directly* and beyond (tight) cognitive control, as illustrated by stimulus–response compatibility (SRC) phenomena, such as the *Simon effect* (Simon & Rudell, 1967). In the typical Simon task, stimuli vary on a spatial dimension (e.g., randomly appearing on the left or right) and on a non-spatial dimension (e.g., having different colors). Participants are to respond to the non-spatial stimulus feature by performing a spatially defined response (e.g., pressing a left or right key). Although the location of the stimulus is irrelevant for the response choice, it nevertheless influences response time and accuracy: participants respond faster (and more accurately) when the stimulus location is congruent with the response location than when the stimulus location is incongruent with the response location. This finding suggests that there is a direct interaction between stimulus perception and response planning. The Simon effect is a very robust finding, has been replicated numerous times and has been used frequently as a methodological tool to investigate perception, action, and cognitive control (for general overviews, see Hommel, 2011; Proctor, 2010).

To account for SRC phenomena cognitive theories and computational models of stimulus–response translation typically assume the existence of two translation ‘routes’ (e.g., DeJong, Liang, & Lauber, 1994; Kornblum, Hasbroucq, & Osman, 1990; Zorzi & Umiltà, 1995): a controlled route for processing task relevant stimulus features (e.g., stimulus color) and an automatic route for processing task irrelevant stimulus features (e.g., stimulus location). Both routes end in response codes labeled with the response locations in the task (e.g., ‘left key’ and ‘right key’). When the relevant stimulus feature and irrelevant stimulus feature both activate the same response code, processing is facilitated yielding smaller reaction times; conversely, when they activate different response codes, processing is interfered yielding larger reaction times.

Although these process models are typically able to fit behavioral data quite well, they leave some issues—relevant for understanding the interaction between perception and action from a representational perspective—unaddressed. The three issues we focus on here are: (1) a common characteristic of these models is the use of response codes with intrinsic connotations (e.g., direction or location) as exemplified by their respective labels (e.g., ‘left key’). The question then arises, how the cognitive system has acquired this knowledge and how the knowledge is grounded in the real world. In various empirical studies (e.g., Riggio, Gawryszewski & Umilta, 1986; Guiard, 1983; Hommel, 1993), it has been shown that this connotation depends on the task context. So, how may situation-specific meanings of motor actions emerge? (2) How and why are some task irrelevant features connected to response codes with an automatic route? In these models automaticity is simply assumed if stimuli and responses are ‘similar’ (e.g., “have dimensional overlap”, Kornblum, et al., 1990; Kornblum, Stevens, Whipple & Requin, 1999) without providing a theoretical rationale or a concrete mechanism accounting for similarity. Finally, (3) various empirical studies (e.g., Haazebroek, van Dantzig & Hommel, 2013; Hommel, 1993) show that the task context may substantially influence both the occurrence and direction of SRC effects.

An influential model that addresses the influence of task context on SRC effects explicitly in the context of the Stroop task (Stroop, 1935) has been developed by Cohen, Dunbar and McClelland (1990). This connectionist model contains two sets of input units (i.e., red and green ink color units; and RED and GREEN word units), a set of intermediate units, and a single set of output units (“red” and “green”). Input units are activated and activation propagates through connections, via the intermediate units, towards the output units. This model too contains two pathways: one for color-naming and one for word-reading. When the input to the model consists of a congruent stimulus, such as the word RED in ink color red, activation propagates, through both pathways, towards the same output unit “red”. If the stimulus is incongruent, however, (e.g., the word GREEN in ink color red), activation is propagated to both the “red” and “green” output units. Crucially, a set of additional input units, so-called task demand units, are also connected to the intermediate units. Their activation modulates which pathway is more dominant in the activation of the output units. By activating the ‘word-reading’ task demand unit, the word is the task-relevant feature and the ink color task-irrelevant, and vice versa when input is given to the ‘ink color’ task demand unit. This early model has been successful in simulating a variety of behavioral data on the Stroop effect and made a substantial contribution in the modeling of attention. The model is strictly feedforward, and, in line with the PDP tradition (e.g., Rumelhart, Hinton, & McClelland, 1986) considered modular. Indeed, Cohen, et al. (1990) make it clear that they assume that ‘some other module’ provides the input for the task demand units. Moreover, the authors state it has not been their focus to consider how task interpretation occurs or how the allocation of attention is determined.

This aspect, and the issues mentioned above, we address explicitly in the model we propose: HiTEC, a connectionist model in the same PDP tradition as the models by Cohen, et al. (1990) and Zorzi and Umiltà (1995). HiTEC is structured differently, however, containing so-called ‘feature’ units that are *shared* by perception and action control. Also, the connections between these ‘feature’ units and output units are not fixed, but are acquired through ideomotor learning (Hommel, 2009; James, 1890; Lotze, 1852; Stock & Stock, 2004), which in turn creates stimulus–response similarity through sharing of feature codes. Moreover, the model is not strictly feedforward and in that sense promotes a more dynamic and integrative perspective on perception and action than the modular views of traditional connectionist models. The units in HiTEC do not originate from a specific task or experimental paradigm, they rather relate to different levels of representation as suggested by the Theory of Event Coding (TEC, Hommel, Müsseler, Aschersleben & Prinz, 2001). TEC aims at capturing the interaction between perception and action in terms of representations and processes that fit well within common neural constraints, and the way this interaction is mediated by cognitive control. HiTEC in turn is based on TECs core principles: (1) a level of common representations, where stimulus features and action features are coded by means of the same representational structures; (2) stimulus perception and action planning are considered to be similar processes, both involve activating these common representations; (3) action features refer to the perceptual consequences of a motor action; when an action is executed, its perceptual effects are associated with the active motor code; one can subsequently plan an action by anticipating the perceptual features belonging to this motor code; and (4) representations are considered to be “intentionally weighted” according to the task context (Fagioli, Hommel, & Schubotz, 2007). HiTEC extends and further specifies TEC in that it includes a task level and procedures regarding task set implementation and action–effect learning—cognitive control, that is, which was not the main target of the original TEC. These additions enable HiTEC to account for a series of key experimental findings on the interaction between perception and action, ranging from stimulus–response compatibility to action–effect learning, in a unitary architecture and at a level of specificity that allows for computer simulation and concrete empirical testing.

In earlier work, we have developed some of the modules and principles integrated in HiTEC, and for instance shown how basic stimulus–response translation may be used for improving cognitive robotics (Haazebroek, van Dantzig, & Hommel, 2011). We showed that the basic Simon effect follows naturally from HiTECs architecture and related this to the notion of affordances in robotics (Haazebroek, et al. 2011). In the following we provide a comprehensive and detailed description of these and other modules, now integrated into the HiTEC model, their interrelationships, and their dynamics. We also present simulation results of five different SRC paradigms. Some of these paradigms have been modeled before using PDP models (e.g., Simon and Stroop tasks, as discussed above), and so we do not claim that our model is unique in accounting for these effects in principle. What we do claim, however, is that HiTEC provides a theoretical rationale and a mechanistic basis explaining how and in particular why SRC occurs. Hence, or a main goal is not efficiency or accuracy in simulating particular outcomes but in gaining theoretical insight through modeling. Crucially, the fifth simulation, which targets the findings of Hommel (1993), demonstrates HiTECs ability to account for the inversion of the Simon effect, which has not been modeled before and clearly shows the role of task context in action control and the flexibility required to capture this effect. To summarize, the focus in this article is not on predicting specific aspects of RT distributions or optimizing particular model parameters. Rather we aimed to provide a proof of principle that a minimal set of strictly theoretically derived representational and interactive processing principles is sufficient to characterize and explain different kinds of perception–action interactions demonstrated in various empirical studies. Moreover, we were interested to see whether the emergence of the cognitive structure generating such interactions can be made part of the modelling process; in other words, we aimed at modelling both the *generation* of cognitive representations and the *processing dynamics* they engage in.

In the next section we discuss our design considerations and present the HiTEC model. Then we present the results of five simulations demonstrating HiTECs dynamics in various SRC paradigms. Finally, in the discussion, we draw a more elaborate comparison between HiTEC and existing models focusing on the three issues discussed above.

## HiTEC model

In this section we describe the HiTEC connectionist model in full detail. We start out with discussing the general cortical architecture of the primate brain and our general connectionist modeling approach. Then we describe the specific HiTEC architecture, followed by its computational implementation. Finally, we discuss how HiTEC allows for simulating behavioral studies and describe its general dynamics during perception and action planning.

### Levels of representation and interactivity in the cerebral cortex

Neurons in the primate cortex appear to be organized in numerous interconnected cortical areas. It is commonly assumed that this organization allows the brain to encode perceived objects in a distributed fashion. That is, different features seem to be processed and represented across different cortical areas (e.g., Cowey, 1985; DeYoe & Van Essen, 1988), coding for different perceptual modalities (e.g., visual, auditory, tactile) and different dimensions within each modality (e.g., visual color and shape, auditory location and pitch). Sensory areas contain neurons that are responsive to specific sensory features (e.g., a specific color or a specific visual location). Areas in the motor cortex contain neurons that code for more or less specific movements (e.g., the muscle contractions that produce the movement of the hand pressing a certain key). Higher up in the processing stream there are cortical areas containing neurons that are receptive to stimulation from different modalities. In effect, they are considered to integrate information from different senses and modalities. Finally, the prefrontal cortex contains neurons that are generically involved in cognitive control of various tasks (Duncan & Owen, 2000). These levels of representation form the basis of the HiTEC model architecture. Crucially, cortical areas for different levels of representation are not only interconnected by feedforward connections but there are also dense neural pathways from centers of higher brain function back into perception centers (Braitenberg & Schüz, 1991; Young, 1995) suggesting top-down influence of higher level areas on processing within lower level areas (e.g., Prinz, 2006). This aspect of reciprocal connectivity between various levels of representation is central to the HiTEC connectionist model.

### Connectionist approach

In line with the interactive activation connectionist modeling approach (PDP; e.g., Rumelhart, et al., 1986), information processing occurs through the interactions of a large number of interconnected elements called units. Each unit may stand for a group of neurons (i.e., localist coding). Units are organized into higher structures representing cortical layers. Each unit has an activation value indicating local activity. Processing occurs by propagating activity through the network; that is, by propagating activation from one unit to the other, via weighted connections. When a connection between two units is positively weighted, the connection is excitatory and the units will increase each other’s activation. When the connection is negatively weighted, it is inhibitory and the units will reduce each other’s activation. Processing starts when one or more units receive some sort of external input. Gradually, unit activations rise and propagate through the network while interactions between units control the flow of processing. Some units can be designated output units. When activations of these units reach a certain threshold the network is considered to produce the corresponding output(s).

### HiTEC architecture

**Fig. 1:**
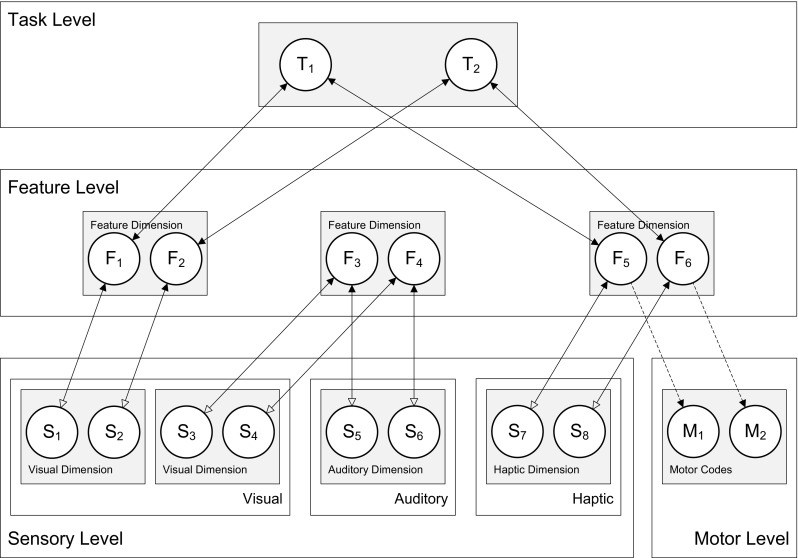
General computational structure of HiTEC. Codes are contained in layers at various levels, and are connected by excitatory connections. *Solid lines* denote fixed weights, *dashed lines* are connections with learned weights. Sensory codes receive modulated excitatory input from feature codes, denoted by the *open arrows*. Note that feature code–motor code associations are one-way connections and that feature code–task code connections are non-modulated both ways

In line with the cortical representation principles and interactivity discussed above, HiTEC has a multiple-layer architecture (see Fig. 1) and recurrent interactions at multiple levels, including feedback to lower level units. In HiTEC feedforward and feedback interactions are cooperative and lateral interactions (i.e., within layers) are competitive (see also Murre, Phaf & Wolters, 1992; van Dantzig, Raffone & Hommel, 2011). The HiTEC neural network is composed of excitatory and inhibitory neural units in each layer. The coding functions are implemented as excitatory units. The inhibitory units are only involved in lateral competitive interactions; by contrast, the excitatory units can receive inputs from and send outputs to associated units in other layers, yielding cooperative interactions. Within each layer inhibitory units are activated by an associated excitatory unit and propagate inhibition to the excitatory units that implement other codes in the same layer.

We now first outline the general model architecture, and then describe the model behavior and the computational specification of the network units. HiTECs general architecture contains sensory layers, feature layers, a task layer and a motor layer, as depicted in Fig. 1. Each layer resembles a cortical circuitry and contains codes implemented as excitatory connectionist network units as described above.

Note that the connection weights can be different (asymmetrical) for corresponding ‘forward’ and ‘backward’ connections (e.g. different weights for the connection from feature codes to task codes, and the reciprocal connection from task codes to feature codes). The different codes (and related units) are characterized as follows.

#### Sensory codes

In HiTEC, different perceptual modalities (e.g., visual, auditory, tactile, proprioceptive) are distinguished and different dimensions within each modality (e.g., visual color and shape, auditory location and pitch) are processed and represented in different sensory layers. Each sensory layer contains a number of sensory codes that are responsive to specific sensory features (e.g., a specific color or a specific location in the visual field). Sensory codes receive external input and feedback activation from feature codes. Crucially, the responsiveness of sensory coding units is modulated by connected feature coding units. This is realized by making the inputs from feature units to a sensory coding unit dependent on that sensory coding unit’s activation, which is primarily determined by its external stimulation. This way, a sensory coding unit cannot become highly active by mere top down input, which would be the equivalent of a hallucination.

#### Motor codes

The motor layer contains motor codes, referring to more or less specific movements (e.g., the movement of the hand pressing a certain key or producing a verbal utterance). Although motor codes could also be organized in multiple layers (e.g. reflecting different body parts), in the present version of HiTEC we consider only a single basic motor layer with a set of motor codes. Motor codes are activated by feature codes. When the activation level of one of the motor coding units reaches a set response threshold, the motor code is assumed to be selected and executed. Subsequent *action effects in the environment* are presented to the sensory coding units allowing the model to learn action–effect contingencies (see Ideomotor Learning below). Note that our present account of motor information represents a dramatic simplification. Movements are unlikely to be represented by coherent, encapsulated motor programs (as considered by Keele, 1968) but, rather, in a rather complex, distributed fashion (Hommel & Elsner, 2009; Wickens, Hyland, & Anson, 1994). However, this simplification does not affect our main arguments and it helps keeping the model and its behavior reasonably transparent.

#### Feature codes

TEC’s notion of feature codes (Hommel, et al., 2001) is captured at the feature level by codes that are connected to and thus grounded in both sensory codes and motor codes. Crucially, the same (distal) feature code (e.g., ‘left’) can be connected to multiple sensory codes (e.g., ‘left proprioceptive direction’ and ‘left visual shape’). Thus, information from different sensory modalities and dimensions is combined in one feature code representation. It is assumed that feature codes arise from regularities in sensorimotor experience, presumably by detecting co-occurrences of sensory features. Since feature codes connect to both sensory codes and motor codes, they can be considered common codes in the sense of Prinz (1990), subserving both stimulus perception and response planning. When a certain feature code is used to represent a task stimulus and this same feature code is also used to represent a task response, the resulting code overlap may result in compatibility effects. Such compatibility effects are demonstrated in the simulations discussed in the next section.

#### Task codes

The task layer contains generic task codes that reflect alternative stimulus–response combinations resulting from the task context. Different task codes reflect different stimulus–response choice options within the task context. Task codes connect bi-directionally to feature codes, both the feature codes that represent stimuli and the feature codes that represent responses, in correspondence with the current task context. Note that task codes themselves are considered task-generic (i.e., labeled ‘T_1_’, ‘T_2_’ et cetera) representations that are re-used across multiple tasks, in line with findings of ad-hoc recruitment of neurons in PFC for task-generic decision making (Duncan & Owen, 2000); the meaning of a task code is different for each task and completely derives from its connections with specific feature codes.

### Basic model behavior

The presentation of a stimulus is simulated by feeding external input to the appropriate (excitatory) sensory codes relating to the various stimulus features (e.g., its location, color, auditory tone et cetera). This results in a gradual increase of their activation level, which is translated into output to feature codes. Thus, activation flows gradually from sensory codes to (stimulus related) feature codes to task codes to (response related) feature codes to motor codes. Once a motor code is activated strongly enough it is assumed to lead to the execution of a motor response to the presented stimulus. The gradual passing of activation between codes in different layers along their connections is iterated for a number of simulation cycles, which allows for the simulation of reaction time (i.e., number of processing cycles from stimulus onset to response selection). Crucially, activation also propagates back from task codes to stimulus related feature codes that in turn modulate the sensitivity of sensory codes, thereby rendering an integrated processing system with both feedforward and feedback dynamics rather than a serial stage-like processing mechanism.

### Ideomotor learning

In HiTEC, following TEC, connections between feature codes and motor codes are not fixed but learned according to the ideomotor principle (Hommel, 2009; James, 1890; Lotze, 1852; Stock & Stock, 2004). This principle states that when one executes a particular action and perceives the resulting effects in the environment, the active motor pattern is automatically associated to the perceptual input representing the action’s effect. Based on these action–effect associations, people can subsequently plan and control a motor action by anticipating its perceptual effect.

In similar vein, learning in HiTEC is done by first alternately activating motor codes, not unlike the exploratory movement behavior patterns of newborn infants (motor babbling, see Meltzoff & Moore, 1997 for an overview) or complete novices at a new task. When a motor code reaches a threshold of activation, we assume that the response is executed, resulting in perceivable changes in the environment (action effects). In HiTEC these action effects are perceived by stimulating the respective sensory codes; activation is subsequently propagated from these sensory codes towards feature codes (cf. Elsner & Hommel, 2001). Finally, associations are learned between these feature codes and the executed motor code. During subsequent stimulus–response translation these associations enable activation of the appropriate motor action by activating the associated feature codes. Thus, a motor action can indeed be selected by ‘anticipating its perceptual effects’ using feature codes. Crucially these same feature codes are also used in stimulus perception. This, in turn, sets the stage for compatibility effects which are the main focus of the current work.

### Task internalization

In behavioral experiments both stimuli and responses can have a variety of features. The task context dictates which of these features are relevant (i.e., the features to look for and to discriminate) and which are irrelevant. In HiTEC, a task instruction is implemented by connecting feature codes and task codes according to the actual task rules in terms of stimulus features and response (i.e., action effect) features. This procedure allows the task instruction to be readily internalized in a principled manner. An example task instruction “when you hear a high tone, press the left key” can be implemented as connections from ‘High’ to ‘T_1_’ and from ‘T_1_’ to ‘Left’ and ‘Key’. During the subsequent stimulus–response translation, these connections modulate the responsiveness of feature codes to bottom-up input from stimulated sensory codes and through these connections activation is propagated towards feature codes associated to the proper motor responses in accordance with task demands (cf., Miller & Cohen, 2001). This way, appropriate goal oriented behavior can take place within a certain task context.

### Computational implementation

HiTEC codes are implemented as (excitatory) neural network units, characterized by an activation level. These units, which may stand for neuronal groups, receive excitatory and inhibitory inputs from other units and background noise. Excitatory inputs can either be voltage independent or voltage dependent, i.e. with a modulatory role dependent on the voltage (‘activation’) of the receiving unit. Indeed, cortical feedback connections are generally voltage dependent, i.e. necessitate a sufficient level of feedforward (stimulus related) synaptic input to be effective. In addition, the activation of the units is characterized by a decay rate, so that in case of absence of any input the activation will decay exponentially towards a resting level. Units in the sensory layers can also receive an external (stimulus related) input. Thus, on every cycle unit activations are updated according to the following equation:

1 $$ A_{i} (t + 1) = \left( {1 - d_{\text{a}} } \right) \times \;A_{i} (t) + \gamma \times \left( {{\text{Exc}}_{i} \times \left( {1 - A_{i} (t)} \right) + {\text{Inh}}_{{{\kern 1pt} i}} \times A_{i} (t)} \right) $$

In this equation, *d*_a_ is the activation decay rate, *A*_*i*_(*t*) is the activation level of unit *i* at time *t*, Exc_*i*_ is the sum of its excitatory input, Inh_*i*_ is its inhibitory input and *γ* is a scaling term. Note that both excitatory and inhibitory inputs are scaled in a way that the unit’s activation may take on any real value between 0.0 and 1.0. The excitatory input is computed as follows:

2 $$ {\text{Exc}}_{{i{\kern 1pt} }} = {\text{ExcVI}}_{{{\kern 1pt} i}} + {\text{ExcVD}}_{{{\kern 1pt} i}} + {\text{Ext}}_{{{\kern 1pt} i}} + {\text{Noise}}_{{{\kern 1pt} i}} \, $$

Here, ExcVI_*i*_ is a voltage independent (‘non-modulatory’) input from other units in the network, which does not depend on the activation of the receiving unit; ExcVD_*i*_ is a voltage dependent input, which is instead dependent on the activation of the receiving units (implicitly related to the membrane potential of receiving neurons). These different excitatory inputs stand for different synaptic currents in cortical networks: feedforward signaling takes place by voltage-independent synaptic currents, and feedback signaling by modulatory voltage dependent currents (e.g., Dehaene, Sergent, & Changeux, 2003; Raffone & Pantani, 2010; Tononi, Sporns, & Edelman, 1992). Ext_*i*_ is input from external stimulation (only for units in the sensory layers) and Noise_*i*_ is a noise term. This noise term is determined by drawing a random value from a Gaussian distribution at each update cycle and for each unit independently. Such noise term is introduced to capture the stochastic background of spiking activity in the cortex (Amit & Brunel, 1997; Grossberg & Grunewald, 1997) and for variance in network activity across simulation trials. The voltage independent input is obtained by calculating the weighted sum of the outputs of all connected units (apart from units where voltage dependent input applies, see below):

3 $$ {\text{ExcVI}}_{i} = \sum\limits_{k} {w_{k}^{ + } F(A_{k} (t))} $$

Here, *w* ^+^ are the positive weights of the connections from other units *k* to unit *i*. The output of a unit is a non-linear function of its activation value, using the following function (Grossberg & Grunewald, 1997; Grossberg & Somers, 1991), with parameters na and qa:

4 $$ F(A_{i} ) = \frac{{A_{i}^{\text{na}} }}{{({\text{qa}})^{\text{na}} + A_{i}^{\text{na}} }} $$

Crucially, the responsiveness of sensory coding units is modulated by connected feature coding units. This is realized by making the inputs from feature units to a sensory coding unit dependent on the sensory coding unit’s activation, which is primarily determined by its external stimulation. This way, a sensory coding unit cannot become highly active by mere top down input. This voltage dependent input from feature coding units to sensory coding units is computed using the following equation (see Tononi, et al., 1992, for a similar computation):

5 $$ {\text{ExcVD}}_{i} = \sum\limits_{k} {w_{k}^{ + } F(A_{k} (t))} \times \frac{{\hbox{max} (A_{i} (t)\, \times (1 - d_{\text{a}} ) - {\text{VT}},0)}}{{1 - {\text{VT}}}}\quad $$

Here, *d*_a_ is the activation decay rate and VT is the voltage threshold. When the sensory coding unit has a (scaled) activation level higher than this threshold, top down input from connected feature coding units is taken into account, rescaled in proportion to the voltage threshold and added to the sensory coding unit’s excitatory input. If the sensory coding unit’s scaled activation level is lower than the voltage threshold, this input is discarded.

Activation of units is competitive, so that coding units within the same layer (sensory layers, feature layers, task layer, or motor layer) inhibit each other. This is computationally realized by the involvement of ‘paired units’. Each of the inhibitory units receive activation from its excitatory paired unit, and propagates inhibition (i.e., their ‘outgoing’ connections are negatively weighted) to all other excitatory units within the same layer. Such inhibition is characterized by non-linearity, i.e. inhibitory units propagate inhibition when they approach a level of activation. This mechanism ensures that within a layer only one unit becomes highly active after a certain number of simulation cycles.

Inh_*i*_ is computed using the following equation:

6 $$ {\text{Inh}}_{i} = \sum\limits_{k} {w_{k}^{ - } F(A_{k} (t))\;} $$

Here, *k* denotes the inhibitory units belonging to any other unit than unit *i* in the layer, and *w* ^−^ are the negative connection weights. The activation of inhibitory units is updated in a similar fashion as the excitatory units, but their input can only be excitatory from the associated paired unit.

#### Connections

Weights between sensory coding units and feature coding units reflect long term experience and are set by hand in HiTEC. Weights of the connections between feature coding units and task coding are also set by hand, closely following the task instruction. Only the weights from feature coding units to motor coding units are learned using Hebbian learning.^1^ Specifically, at the end of each learning trial, all weights are updated (synchronously) according to the following set of equations:

7 $$ w{\kern 1pt}_{jk} (t + 1) = (1 - d_{\text{w}} ) \times w_{jk} (t) + {\text{Act}}_{j} (t)\; \times {\text{Act}}_{k} (t)\; \times \left( {1 - w_{jk} (t)} \right) $$

$$ {\text{Act}}_{j} (t) = \frac{{A_{j} (t) - {\text{LT}}}}{{1 - {\text{LT}}}}\quad {\text{if}}\,A_{j} (t) > {\text{LT}} $$

$$ {\text{Act}}_{j} (t) = 0\quad {\text{if}}\,A_{j} (t) \le {\text{LT}} $$

$$ {\text{Act}}_{k} (t) = \frac{{A_{k} (t) - {\text{LT}}}}{{1 - {\text{LT}}}}\quad {\text{if}}\,A_{k} (t) > {\text{LT}} $$

$$ {\text{Act}}_{k} (t) = 0\quad {\text{if}}\,A_{k} (t) \le {\text{LT}} $$

In these equations, *w*_*jk*_ is the weight from feature coding unit *j* to motor coding unit *k*, the *d*_w_ weight decay rate ensures that only repeated co-activations result in stable weight learning, Act_*j*_(*t*) is a value based on the activation of feature coding unit *j*, Act_*k*_(*t*) is a value based on the activation of motor coding unit *k*, LT is the learning threshold (above which the activation levels of both units must be to engage in weight learning) and *A*_*j*_(*t*) and *A*_*k*_(*t*) are the actual activation levels at time *t* of feature coding unit *j* and motor coding unit *k,* respectively. Note that we rescale the activation of both units to their respective proportion to the learning threshold and that the computed connection weights are bound to vary between 0.0 and 1.0. Also note that there are no weights from motor coding units to feature coding units, so learning is one direction only.

The total number of codes (coding units) and connections varies with the specific instances of HiTEC used for the different simulations. All parameters and weights (when not learned) as used in the simulations are fixed across all model instances. They are listed in the “Appendix”. In general, higher decay rates make units decay faster; lower decay rates keep units very active for a longer period of time. Higher input values for external input and stronger weights between units result in faster activation propagation. Higher voltage thresholds make unit activation to a lesser extent enhanced by top down input; conversely, lower voltage thresholds lead to earlier and stronger influence of top down modulation on unit activation. Stronger weights between excitatory and inhibitory units strengthen the lateral inhibition mechanism. As a result, they reduce the time required to settle the competition between the units within a shared layer, after which only one unit remains strongly activated. Lower weights, conversely, lengthen this time to convergence.

Parameters were thus chosen to enable feedforward propagation of activation in the network to capture in an idealized implementation neurally plausible properties of temporal integration of signals and non-linear response properties of excitatory and inhibitory neurons (Dehaene, et al., 2003; Wilson & Cowan, 1972; Grossberg & Somers, 1991; Wang, 1999). The strength of voltage-dependent top-down connections was chosen to enable their modulatory action without causing spurious activations in the absence of sensory input (Raffone & Pantani, 2010; Tononi, et al., 1992). Taken together, such connection strength and temporal integration and decay parameters were also chosen to avoid the saturation of the activation level of the excitatory units with feedforward input, so to enable sensitivity of such activations to recurrent interactions involving multiple units and top-down signals over time, in dynamic balance with lateral (intra-layer) inhibitory interactions.

Note that our ambition for HiTEC has not been to search for specific parameter values to optimally fit specific data distributions. We rather set out to provide a proof of principle as to how neurally inspired representations and connectivity may realize stimulus–response translation while addressing critical theoretical issues such as action control, automaticity and coping with task context.

### Simulating behavioral studies

To model a behavioral study or experimental paradigm in HiTEC, a specific instance of the HiTEC model is constructed with layers, codes (coding units) and connections that match the stimulus, response, and task characteristics of the simulated experiment. Crucially, connections between feature codes and task codes are set to reflect the exact task instructions.

In each simulation there are two phases: first, action effects are learned, reflecting the period in which the participants get acquainted with the keypresses and their effects, which is commonly part of behavioral experiments. In this learning phase, we allow the model a set number of learning trials (i.e., 20 learning trials, similar to the number of learning trials in the various behavioral experiments) to acquire the associations between feature codes and motor codes. Note that when a motor code is executed, the changes in the environment (i.e., its action effects) are presented by supplying input to the sensory codes. Propagating activation towards feature codes (i.e., for 50 cycles) allows the model to subsequently learn the feature code–motor code associations. Note that in behavioral experiments the task context influences ideomotor learning. In similar vein, in HiTEC, the task related representations and connections are already in place during the learning phase. This mere activity biases the learning process which results in various behavioral phenomena as we will discuss in the next section.

**Fig. 2:**
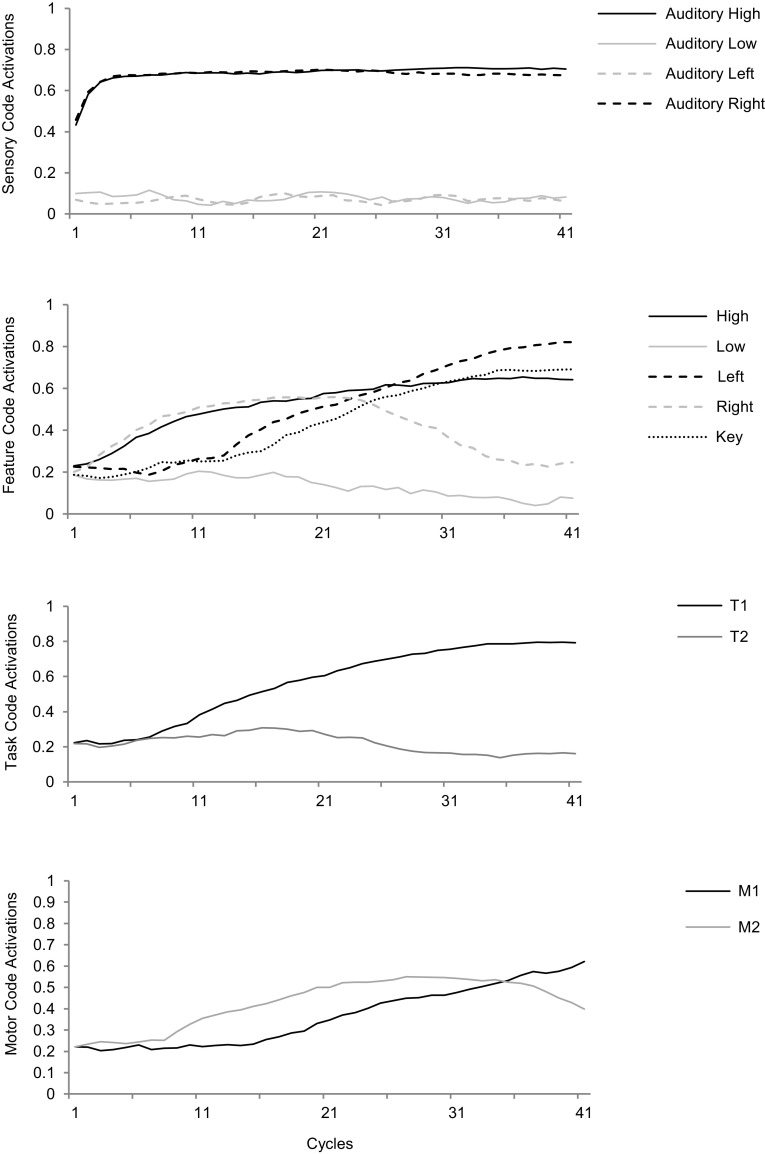
An illustration of the model dynamics: stimulus-induced activation propagates from sensory to feature codes, involving task and motor codes. The *figure* shows incongruent trial in the Simon task (see below for more details). The high-pitched, right stimulus tone feeds into the sensory codes ‘Auditory high’ and ‘Auditory right’ and activation propagates towards feature codes. Due to prior action–effect learning, feature code ‘Right’ eventually propagates activation to motor code ‘M_2_’, while activation also propagates from the ‘High’ feature code towards the task codes, resulting in a more strongly activated ‘T_1_’ and less strongly activated ‘T_2_’. ‘T_1_’ further propagates activation towards feature code ‘Left’, whose activation level eventually exceeds the level of ‘Right’. At the same time activation propagates from ‘Right’ to the associated motor code ‘M_1_’, which eventually exceeds the activation level of ‘M_2_’ and reaches the response threshold. At that point, feature codes ‘Left’ and ‘Key’ are also highly activated. Note that these feature codes resemble the action effect of the produced response

In the subsequent, experimental, phase the model is presented with various stimuli by supplying input to specific sensory coding units. Gradually, activation spreads across all the involved coding units in the various network layers. The trial is terminated at the selection of a motor response and the reaction time is determined based on the number of cycles between stimulus onset and response selection. This enables comparing simulated reaction times with reaction times of human participants in behavioral experiments, but the model also provides insights into the dynamics of stimulus–response interactions (Fig. 2).

## Simulations

We simulated five key behavioral experiments on stimulus–response processing using the HiTEC model. Taken together, our five simulations demonstrate that HiTEC can account for ideomotor learning (Elsner & Hommel, 2001), response-effect compatibility (Kunde, Koch, & Hoffmann, 2004), stimulus–response and stimulus–stimulus compatibility, and the dependency of stimulus-responsive facts on task intentions (Hommel, 1993). For each simulation we discuss the specific results in the respective section and get back to the general model behavior and the theoretical implications in the Discussion.

### Simulation 1: action–effect learning

Ideomotor theory assumes that action–effect acquisition occurs on-the-fly and Elsner and Hommel (2001) were indeed able to demonstrate that people learn action–effect associations spontaneously. In their Experiment 1, participants responded to a visual cue stimulus by pressing a randomly chosen left or right key. One keypress produced a high tone and the other a low tone, which according to the ideomotor principle should have induced bidirectional associations between motor patterns and tone/pitch representations. In the second phase, participants responded to the tones that previously served as action effects by pressing the same two keys, but now according to a specific instruction (e.g., ‘when hearing a high tone, press the left key’). In one (‘non-reversal’) group, the new instruction heeded the learned relationship between tones and keys, so that the tone that was previously produced by a particular keypress was now signaling that keypress. In another (‘reversal’) group, these relationships were reversed, so that the tone that was previously produced by one keypress was now signaling the other keypress.

If the tone-key combinations in the second phase matched the key-tone combinations from the first phase, participants were faster than if the combinations did not match. This suggests that in the first phase, the tones were spontaneously associated with the keypresses that caused them, and that the emerging associations were bidirectional. Indeed, neuroscientific studies revealed that presenting previously produced action effects activates the corresponding action/motor representations (e.g., Melcher, Weidema, Eenshuistra, Hommel, & Gruber, 2008).

**Fig. 3:**
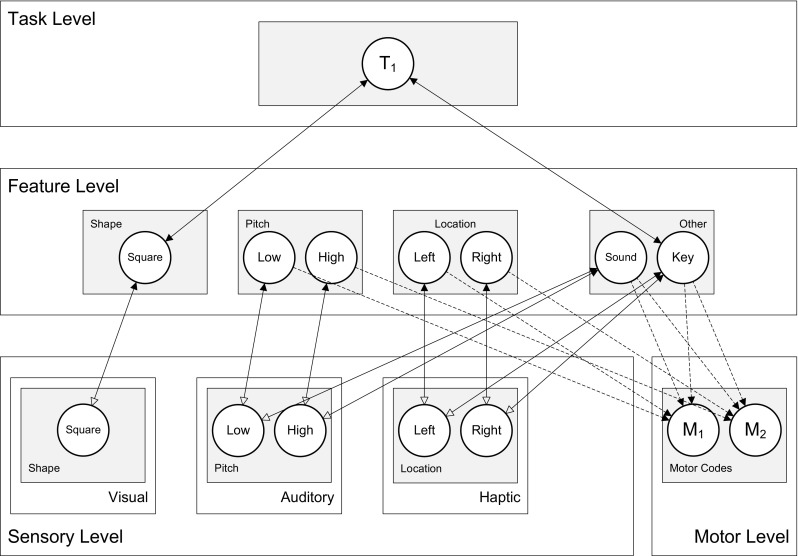
Specific HiTEC model instance for learning trials in Simulation 1. Connections (*dashed lines*) between feature codes and motor codes were learned. Note that in principle any feature code can be connected to any motor code. However, only some of these possible connections actually become (strongly) weighted as a result of the perceived regularities in action effects

To simulate Elsner and Hommel’s (2001) experiment, we created an instance of the HiTEC model with sensory codes for the registration of the visual cue, the auditory pitch levels, and the haptically perceived locations of the keys, with feature codes for the square shape, the pitch levels, the locations, and the ‘Sound’ and ‘Key’ in general,^2^ and with motor codes for the two keypressing actions, as illustrated in Fig. 3. Simulation of the study occurred in two distinct phases: the learning phase and the experimental phase.

During the learning phase motor patterns ‘M_1_’ and ‘M_2_’ were activated alternately and their respective action effects were presented to the model. As a result, associations were learned between the motor codes and the active feature codes.

**Fig. 4:**
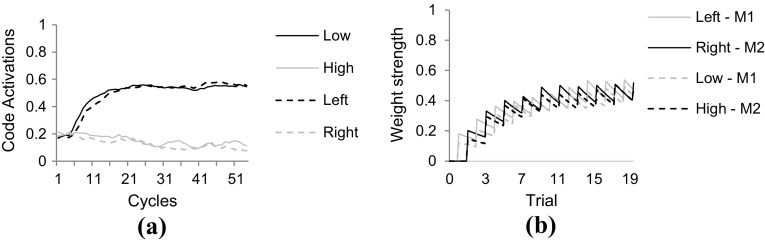
Code activation and connection weight time courses in learning trials of Simulation 1. **a** Activation of feature codes during learning trials as a consequence of perceiving the action effects of the activated motor code. **b** Connections between motor codes and feature codes got gradually stronger over multiple learning trials. Note that feature codes ‘Key’ and ‘Sound’ are omitted from both figures for the sake of clarity

Figure 4a shows a learning trial in which the motor code ‘M_1_’ was activated. This activation led to the simultaneous perception of both a keypress and an auditory tone, resulting in a relatively strong activation of some of the feature codes, including ‘Left’ and ‘Low’. The regularity in combinations of motor actions and their perceivable effects resulted in systematic co-activation of specific motor codes and feature codes. As a consequence, specific motor code–feature code connections were strengthened over time, as is illustrated by Fig. 4b.

**Fig. 5:**
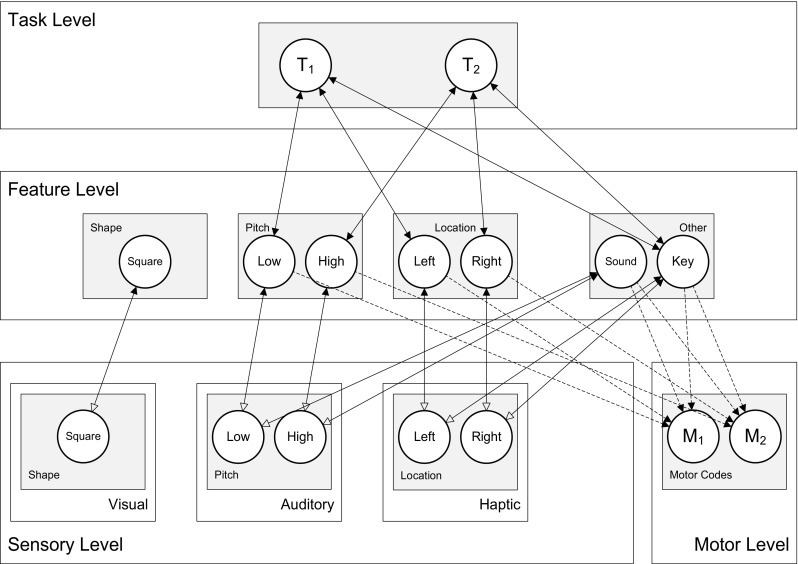
Specific HiTEC model for experimental trials in Simulation 1. Note that only the feature code–task code connections were adapted as compared to Fig. 3, reflecting a new task instruction with the *same* model instance

In the second phase, we let the model instance respond to auditory stimuli with high or low pitch. Note that the change of task (i.e., ‘press a random key’ vs. ‘respond selectively to auditory tones’) was reflected in the change in connections between feature codes and task codes only as illustrated in Fig. 5. The remainder of the model was kept unchanged, most notably the just learned associations between feature codes and motor codes. For this second phase, two copy instances of the model were to respond to stimuli according to two different instructions: the ‘non-reversal’ model instance copy was to respond to the learning-compatible stimuli (i.e., what had been the effect on an action now became the stimulus signaling this action), whereas the ‘reversal’ model instance was to respond to auditory tones with responses that previously produced the alternative tone.

In this second phase, in both model instances, stimulus tones were presented by stimulating auditory sensory codes. Activation subsequently flowed from these sensory codes towards ‘Pitch’ feature codes, task codes and to the ‘Location’ feature codes and the ‘Key’ feature code. Also, activation flowed through the learned associations towards the motor codes. Depending on the stimulus tone, either one or the other motor code reached the response threshold and simulation was terminated. In both conditions, activation flowed from pitch feature codes to task codes to location feature codes, in accord with the task instruction. Simultaneously, however, activation also flowed directly from pitch feature codes to motor codes, along the just acquired action–effect connections. Now, crucially, in the ‘non-reversal’ condition, these connections facilitated processing, whereas these same connections caused interference in the ‘reversal’ condition. As a consequence, in line with the behavioral findings of Elsner and Hommel (2001) the model instance in the non-reversal condition reached the response threshold (29.3 cycles on average) faster^3^ than the instance in the reversal condition (38.5 cycles on average). This simulation demonstrates how HiTECs representations and basic processing principles readily give rise to the observed empirical results demonstrated by Elsner and Hommel (2001).

### Simulation 2: action planning

The observations of Elsner and Hommel (2001) confirm the claim from ideomotor theory that action–effect associations are automatically acquired as demonstrated in Simulation 1. However, this does not yet speak to the further-reaching claim of ideomotor theory that action effects play an important role in the *planning* of intentional actions. Evidence supporting that claim was provided by Kunde, et al. (2004), who showed that choice performance is affected by the compatibility between haptic action effects of the responses proper and novel (auditory) action effects. In their experiment, for one group of participants, responses were followed by a compatible action effect; the loudness of the tone matched the response force (e.g., a loud tone appeared after a forceful key press). In the other group of participants the relationship between actions and action effects was incompatible (e.g., a soft tone appeared after a forceful key press). In both groups, subjects had to respond to a visual cue stimulus by pressing the key softly or forcefully. It was found that the group with action-compatible action effects was faster on average than the group with incompatible action effects. Given that the tones did not appear before the responses were executed, this observation suggests that the novel, just acquired action effects were anticipated and considered in the response-selection process.

**Fig. 6:**
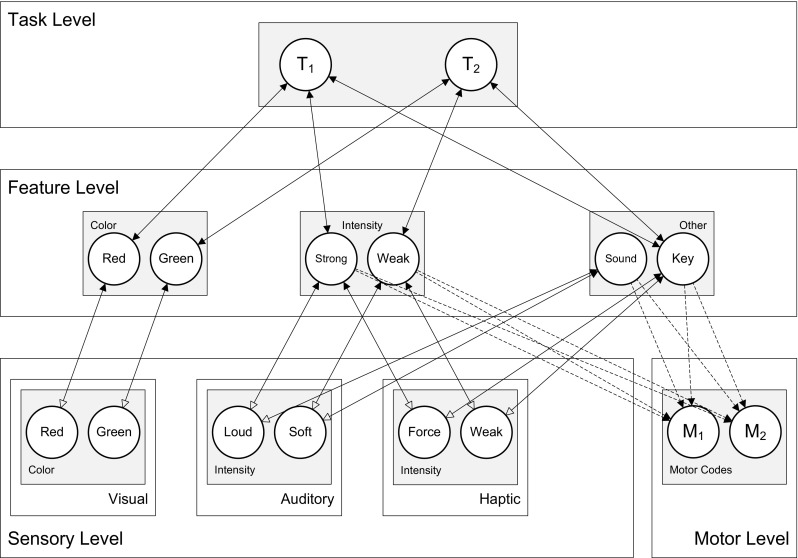
Specific HiTEC model instance for Simulation 2, including feature codes for all stimulus and response features

This effect of response-effect compatibility was simulated in HiTEC. As shown in Fig. 6, the model instance contained sensory codes for the visual colors, auditory intensities and haptic intensities. Motor codes ‘M_1_’ and ‘M_2_’ represented forceful and soft keypresses, respectively. Importantly, to the model these motor codes were not intrinsically forceful or soft but were associated with (and acquired their meaning from) these perceptual characteristics only through learning.

In the HiTEC simulation, this learning was modeled explicitly during a brief learning phase. In this learning phase, motor codes were activated alternately and the model was presented with the appropriate action effects (i.e., haptic intensity and auditory intensity). Crucially, two copy instances of the model received two different action effects, in accord with the two groups in the empirical study: one instance received consistent action effects (e.g., a forceful keypress with a loud tone); the other instance received inconsistent action effects (e.g., a forceful keypress with a soft tone). As a consequence, in the model instance in the consistent condition, the active auditory and haptic sensory codes activated the *same* intensity feature code every time an action effect was presented. This resulted in steady weight increase of motor code–intensity feature code associations. Conversely, in the inconsistent condition, the active auditory and haptic sensory codes activated *different* intensity feature codes every time an action effect was presented. Indeed, a forceful keypress would coincide with a soft tone; and a weak keypress with a loud tone. This resulted in only a mild weight increase of motor code–intensity feature code associations. More specifically, because the task instruction includes pressing keys, the key feature code was connected to the task codes (as depicted in Fig. 6), and because the key feature code was connected to the haptic sensory codes, these haptic sensory codes, when activated, automatically received voltage dependent input from the key feature code. This made them slightly more active than the auditory intensity sensory codes which did not receive such enhancement. As a result the intensity feature code that was activated by the haptic sensory code was also slightly more active than the intensity feature code that was activated by the auditory intensity sensory code, during action–effect learning. As this was the case for every action–effect learning trial, the haptic intensity became the major determinant in the weight learning of connections between ‘Intensity’ feature codes and the motor codes. In more general terms, since the task instruction was already internalized before presenting the learning trials, it biased the learning of connections between feature codes and motor codes. Note that in the simulations discussed in the current work we allow a fixed number of 20 trials of action–effect learning, similar to the number of learning trials in the various behavioral experiments. This, however, does not mean that compatibility effects would vanish when the model would be given unlimited number of trials. As shown in Fig. 4b, the weights stabilize after a number of trials due to the decay rate in weight learning. Moreover, action–effect learning does not depend on imperative stimuli: motor codes are activated and resulting action effects are presented to the model enabling action–effect learning (cf., Herbort & Butz, 2012).

During the actual experimental trials, visual stimuli were presented. This resulted in activation propagation from the visual stimulus codes to the color feature codes to the task codes towards the intensity feature codes and finally the motor codes. Crucially, responding to stimuli required the model to propagate activation along the just acquired intensity feature code–motor code connections. Because the strength of these connections differed for the two different conditions, as a result of task-modulated action–effect learning described above, the model instances differed in their simulated response time. The ‘consistent’ model instance responded faster^4^ (24.0 cycles on average) than the inconsistent model instance (26.0 cycles on average), in line with the empirical data obtained by Kunde, et al. (2004). This simulation demonstrates how HiTEC uses acquired action–effect connections to plan actions and, therefore, shows response-effect compatibility effects. In the current simulation the model is required to respond to stimuli: visual stimuli are presented to the stimuli and the model activates action effects that are associated with the motor codes. It is conceivable, however, that in a free-choice task (e.g., Pfister & Kunde, 2013) the model would also produce a ‘response’ by anticipating action effects. Varying consistency within action–effect associations would then also lead to compatibility effects, as the (in)consistency does not depend on the stimulus but on the ‘internal’ consistency within the action effect (see Pfirster & Kunde, 2013 for a discussion on interpretations in terms of response-effect or effect-effect consistency).

### Simulation 3: Simon effect

A key finding for understanding the interaction between perception and action is the Simon effect. Simon and Rudell (1967) showed that people respond faster to stimuli if the location of the stimulus is compatible with (corresponds to) the response location, even when stimulus location is not task relevant. In the standard Simon task, stimuli with a non-spatial stimulus feature (e.g., auditory pitch) are presented at different locations (e.g., left or right). Participants are instructed to respond to the non-spatial feature by giving a spatially defined response (e.g., pressing a left or right key). Even though the location of the stimulus is not relevant for this task, performance is facilitated when the chosen response corresponds spatially to the stimulus location.

**Fig. 7:**
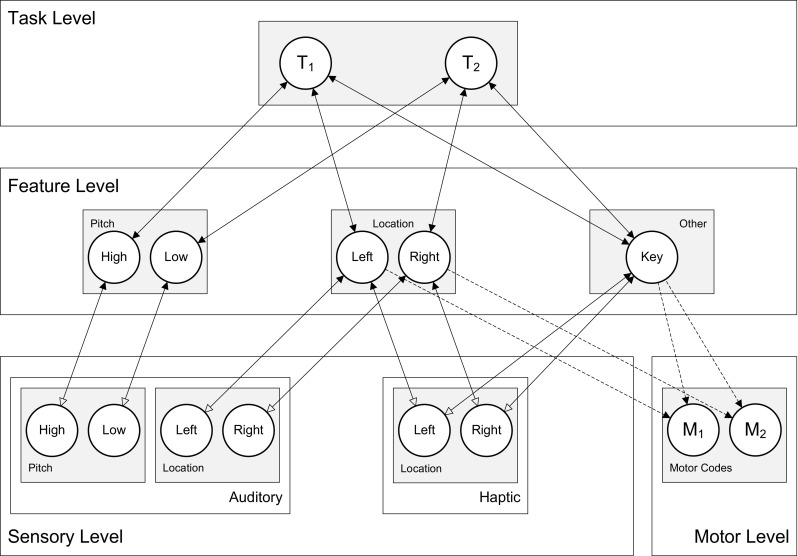
Specific HiTEC model instance for Simulation 3, including feature codes for stimulus pitch and location. Note that location feature codes were used for encoding both stimulus location and response location

The Simon effect was modeled in HiTEC, as shown in Fig. 7, using sensory codes for auditory pitch, auditory locations and haptic locations, feature codes for pitch, location and for ‘Key’ and finally two motor codes, ‘M_1_’ and ‘M_2_’, representing pressing the left and the right key. During the learning phase, ‘M_1_’ and ‘M_2_’ were activated alternately and their respective action effects were presented to the model. As a result, associations were learned selectively between the motor codes and the ‘Left’ and ‘Right’ feature codes.

**Fig. 8:**
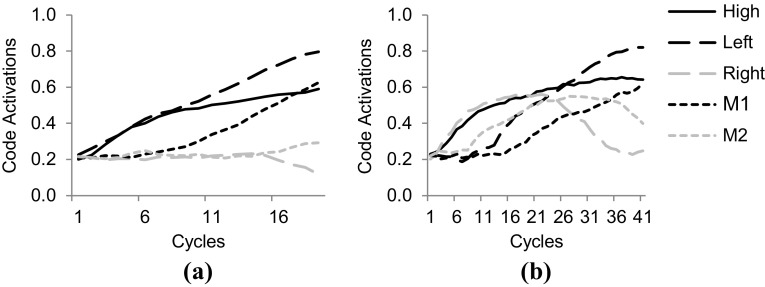
Time courses of feature code and motor code activations in the experimental trials of Simulation 3. **a** Activations during a compatible trial. Here ‘M_1_’ reached response threshold in 19 cycles. **b** Dynamics in a non-compatible trial. Here ‘M_1_’ reached threshold in 41 cycles. Note that activations of other feature codes, task codes and sensory codes are omitted for sake of clarity

In the experimental trials, tones were presented and responded to by propagating activation from sensory codes to pitch feature codes to task codes and to location feature codes and finally motor codes. Crucially, the ‘Left’ and ‘Right’ feature codes were also activated when the tone stimulus was presented on the left or right yielding different dynamics when the tone location coincided (compatible trial) with the key location of the anticipated response than when the tone was on the opposite side (non-compatible trial) as illustrated in Fig. 8.

Essentially, in the compatible condition, the stimulus location already activated the ‘correct’ spatial feature code and thereby sped up response selection. Conversely, in the incompatible condition, stimulus location activated the ‘wrong’ spatial feature code, which also already activated the ‘wrong’ motor code. Meanwhile, however, the stimulus pitch was translated—through the task codes—into the correct spatial feature codes and the correct motor code. This latter pathway overcame the head start due to the overlap-pathway, but the code overlap did slow down the overall translation as reflected in the results. In the compatible condition the HiTEC model was faster^5^ (19 cycles on average) than in the non-compatible condition (38.5 cycles on average) with the neutral condition falling in between (24.5 cycles on average), which is in line with the empirical findings by Simon and Rudell (1967). This simulation demonstrates that implementing a Simon task in HiTEC using common feature codes for stimuli and responses automatically yields the observed compatibility effect.

### Simulation 4: Stroop effect

**Fig. 9:**
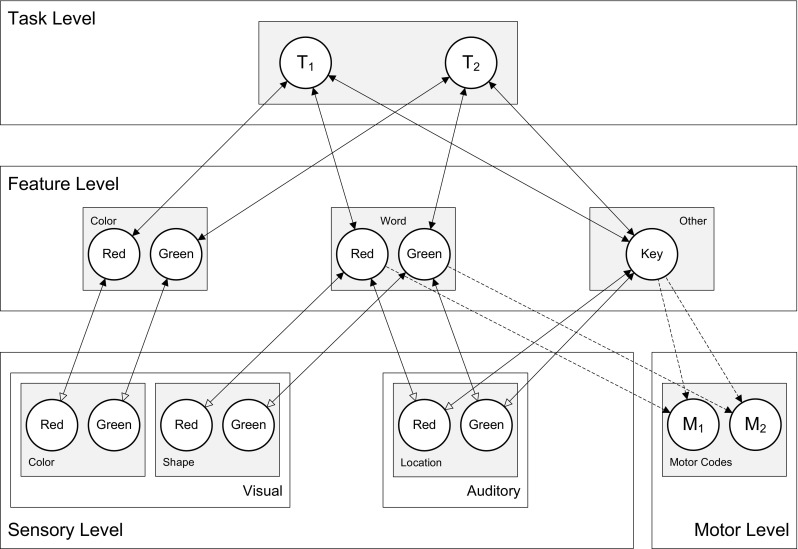
Specific HiTEC model instance for Simulation 4, including feature codes for stimulus colors and words. Crucially, word feature codes were used for encoding both stimuli (i.e., the *color words*) and responses (i.e., the words to name the *ink color*). Connections between word feature codes and motor codes were learned during learning trials (i.e., pronouncing the words). Note that this model structure is in essence identical to the structure of the model used for Simulation 3

As we do not differentiate between perceptual and action stages, one could argue that stimulus–response compatibility and stimulus–stimulus compatibility would need to work similarly in HiTEC. Stroop (1935) showed that if people are instructed to name the ink color of color words, they are slower if the word (e.g., “blue”) appears in an incompatible ink color (e.g., red). This compatibility effect is dramatically reduced if non-verbal responses are required (MacLeod, 1991), suggesting that the task-irrelevant words interfere (at least partly) with verbally naming the colors. Note that this interpretation of the Stroop effect bears a strong resemblance to the Simon effect as the effect is now attributed to incompatibility between a stimulus feature (ink color) and a response feature (verbal sound) (Fig. 9).

In HiTEC the Stroop effect is simulated along the same lines as the Simon effect in Simulation 3. In similar vein, during the learning trials, the model alternately executed ‘M_1_’ and ‘M_2_’, reflecting the ‘physical’ pronunciation of the respective words. The model was subsequently presented with the auditory feedback (i.e., reflecting the perception of this pronunciation) and associations were learned between motor codes and feature codes. During experimental trials, naming ink color of compatible color words benefitted from facilitation whereas naming the color of incompatible color words suffered from interference. Indeed, responses were faster^6^ in the compatible condition (19 cycles on average) and slower (38.5 cycles on average) in the incompatible condition, with the neutral condition falling in between (24.5 cycles on average). This simulation demonstrates that by treating stimulus features and response features similarly, some cases of stimulus-stimulus compatibility may be accounted for using the exact same logic (and processing principles) as for stimulus–response compatibility. In HiTEC, this results in identical simulations.

### Simulation 5: inverting the Simon effect

Hommel (1993) demonstrated that the Simon effect as described in Simulation 3 can be ‘inverted’ by changing the task instruction only. In this study participants responded with left or right keypresses to the high vs. low pitch of tones which were presented left or right. When a key was pressed a flash light was presented on the opposite side of the keypress. One group was instructed to “press the left/right key” in response to the low/high pitch of the tone, whereas another group was instructed to “flash the right/left light” in response to the low/high pitch. In other words, all participants carried out exactly the same movements in response to the same stimuli, but one group did that “in order to press the keys” while the other did it “in order to flash the lights”. This seemingly minor manipulation had a major impact on the Simon effect. Whereas the Key group showed a standard Simon effect with faster responses for spatial correspondence between tones and keys, the Light group showed the opposite effect: faster responses for spatial correspondence between tones and lights. This observation demonstrates the crucial role of task instruction in stimulus and response coding and, more generally, in perception and action planning.

**Fig. 10:**
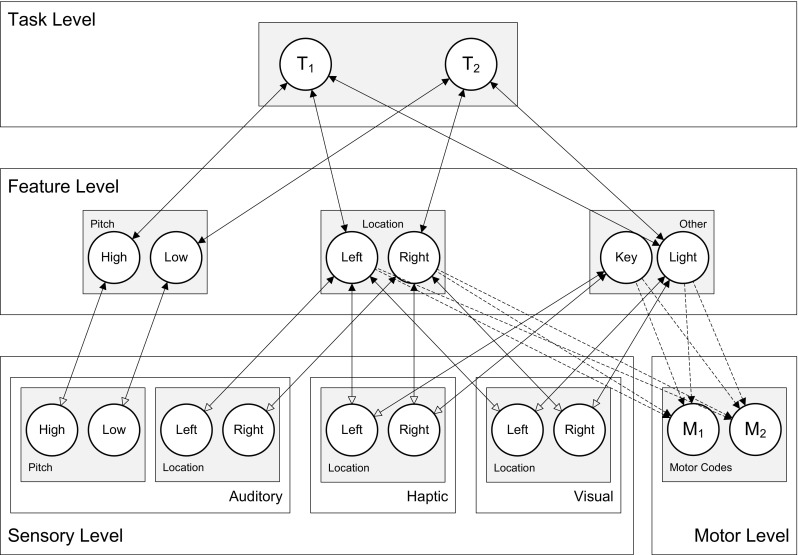
Specific HiTEC model instance for Simulation 5. Shown is the model instance for the Light condition. The Key condition differed only in the connections from ‘T_1_’ and ‘T_2_’ to ‘Key’ instead of ‘Light’. Connections between feature codes and motor codes (*dashed*) are learned

**Fig. 11:**
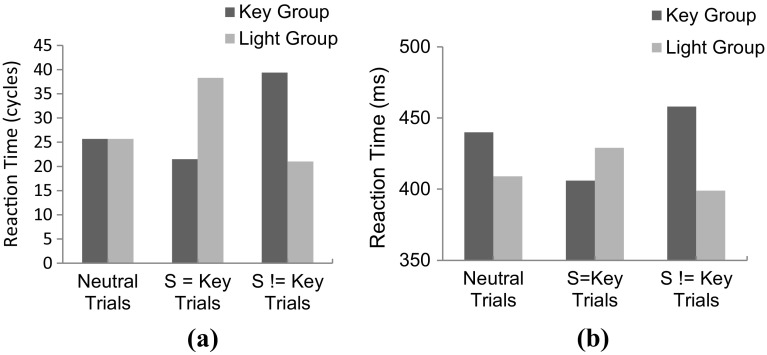
Results of Simulation 5 **a** compared with behavioral data (**b**, adopted from Hommel, 1993). The horizontal axis denotes the various congruency levels

The empirical study was simulated in HiTEC using two instances of the model. One instance was configured according to the Key instruction, the other to the Light instruction. The latter condition is depicted in Fig. 10. Note that the difference in task instructions was reflected in the task connections alone. Crucially, in the ‘Key’ model instance the mere connections between ‘Key’ and the task codes enhanced the processing of haptic locations. In contrast, in the ‘Light’ model instance, the connections between ‘Light’ and the task codes enhanced visual locations. This specific wiring biased the action–effect learning and the direction of the compatibility effect during subsequent experimental trials. The results^7^ are illustrated in Fig. 11. Here, the ‘Key’ model instance showed fastest responses in the congruent stimulus-key condition (21.5 cycles on average), intermediate response time in the neutral condition (25.7 cycles on average), and slowest responses in the incongruent stimulus-key condition (39.4 cycles on average). In contrast, for the ‘Light’ model instance these results were inverted: fastest responses in the incongruent stimulus-key condition (21.0 cycles on average), intermediate response time in the neutral condition (25.7 cycles on average), and slowest responses in the congruent stimulus-key condition (38.3 cycles on average). Together these results yield a pattern similar to the empirical findings reported by Hommel (1993). Note that in the behavioral study additional factors were at play that further influenced the results yielding a non-symmetrical pattern. These factors include possible individual problems with an unfamiliar instruction, but more importantly another difference between key and light conditions: in the key condition not only the goal (key location) was compatible or incompatible with the stimulus, but also the anatomical location (i.e., hand). In the light condition, only the goal (light location) was compatible or incompatible with the stimulus. This may have led to different patterns in both reaction times and error rates (see Hommel, 1993 for a detailed discussion). The notion of anatomical location is not modeled in the current simulation, hence to the model the key and light conditions are completely symmetrical whereas this is not the case for human participants. Overall, this simulation demonstrates that the basic principles of HiTEC allow a task to be implemented in a way that stimuli and responses are encoded flexibly and even ‘automatic’ aspects of stimulus–response translation can be modulated by the task.

## Discussion

In HiTEC the link between perception and action involves representations on multiple levels in a connectionist network, through the interplay of feedforward and feedback connections. Stimuli are presented by feeding external input to sensory codes. Responses are considered to execute when a motor code reaches the activation threshold. Stimuli are translated into responses via an interactive process in the network, with gradual feedforward and feedback propagation of activation in it. Rather than a sequential stepwise process from sensory codes through intermediate representations to response codes, all representations at all levels cooperate and compete and together converge to a response outcome. Crucially, representations at higher levels modulate representations at lower levels. This allows both for direct interaction between perception and action representations and modulation by the task context. Although the rather simple HiTEC model is not intended as a detailed neuroscientific model, it might be worth noting that its components, as well as their connectivity, do map in a gross way onto specific neural systems. The network architecture follows the same general form as more neurobiologically oriented models of visual attention and object selection do (e.g., Deco & Rolls, 2004).

More specifically, our approach is in line with the *integrated competition hypothesis* (Duncan, Humphreys, & Ward, 1997). This hypothesis proposes that visual attention results from competition in multiple brain systems and rests on the three following principles. First, different objects are considered to compete for activation within multiple brain systems. Second, although this competition takes place in multiple brain systems, it is integrated between these systems in such a way that units responding to the same object in different brain systems support each other’s activity, whereas units responding to different objects compete. Finally, competition is considered to be directed on the basis of relevant object properties based on the current task demands. Duncan, et al. (1997) suggest top-down neural priming as a possible control mechanism. HiTEC could be considered both a generalization and specification of this hypothesis. Due to the common coding nature of feature codes, not only visual attention but also action anticipation (and thus action control) are considered to compete for activation, hence generalizing the scope of the integrated competition account. HiTEC further specifies a possible method of directing this competition using task set connections rather than priming. Moreover, HiTEC explicitly addresses how the task instruction could implement such a task set and how task instruction could influence both perception and action planning.

Our notion of interactive processing with mutual influences among multiple subsystems is shared by the early selective action model of Ward (1999), who proposes that action plans may bias selective perceptual processing towards relevant objects (see Hommel, 2010). Like in HiTEC, selected representations receive external input and activation gradually spreads among various units coding through the reciprocal connections converging to a selected object and action. Task context is encoded by priming the units that represent the task-relevant object and/or action feature, which biases the competition between representations accordingly. Like HiTEC, and unlike most other perception–action models, the selective action model can thus account for effects of action planning on perception and attention (e.g., Wykowska, Schubö, & Hommel, 2009).

The most important difference between Ward’s (1999) model and HiTEC concerns the model architecture: Given the emphasis on reaching and grasping, Ward distinguishes explicitly between a ventral ‘what’ and a dorsal ‘where’ pathway (Milner & Goodale, 1995). While this distinction can (and eventually should) be integrated with HiTEC (Hommel, 2010), TEC and HiTEC are mainly concerned with the “ventral” branch of this processing structure, hence, with perception–action interactions at a common coding level of feature representations. Another major difference between the models refers to how a task is internalized. In Ward’s model, a single task rule (e.g., ‘grab the red object’) is set by supplying additional external input to a selection of codes (e.g., ‘red’ and ‘grab’) which then biases the processing of the stimulus input. That is, stimulus presentation and task instruction occur simultaneously and use the same mechanism of applying external input. In HiTEC, task context is internalized instead by interconnecting feature codes and generic task codes based on the task instruction. Then, these connections automatically modulate the propagation of activation resulting from stimulus presentation. Crucially, this allows HiTEC to internalize multiple task rules at the same time that compete during subsequent stimulus–response translation effectively allowing for (minimalistic) decision making, whereas Ward’s model is focused on executing one specific task (e.g., ‘grab the red object’) at a time depending on the codes that receive additional input bias during stimulus processing. Indeed, Ward states that these external inputs are meant to result from decisions that are made outside the scope of this model, which is much in line with Cohen, et al. (1990). The Ward model, however, does allow for selective attention for targets among distractors in the stimulus display, an aspect of attention that HiTEC currently lacks. Finally, although Ward, in accord with our approach, aimed at addressing the interaction between perception and action, his model assumes the implications for action of a given object by fixed connections between given object features (e.g., vertical object orientation) and given, rather specific, actions (e.g., vertical grasp). This connection between perception and action planning is addressed more generically and more explicitly in HiTEC using the notion of common codes and ideomotor learning.

Accordingly, although HiTEC shares some core assumptions with the Selective Action Model, we consider the former as much broader than the latter, which also holds for the recent extension of the selective action model by Botvinick, Buxbaum, Bylsma, and Jax (2009). Moreover, none of these two versions of the selective action model addresses empirical findings of automaticity (i.e., stimulus–response compatibility), which is a major drawback for models targeting the interaction between perception and action. Let us now turn to the three major questions that have guided our study: how can actions acquire situation-specific meanings, how does automatic stimulus–response translation emerge, and how does task context modulate stimulus–response processing?

### Meaning of action

Action control in HiTEC is based on the ideomotor principle, which addresses both the acquisition of action–effect associations and the use of these associations in action planning. Simulation 1 addresses how novel action-contingent perceivable effects are (spontaneously) associated to the actions that yield these effects. Simulation 2 demonstrates how the (internal) consistency of these effects influences the representations of these effects. As action–effect learning depends on the activation of both motor codes and feature codes, the consistency of feature code activation has consequences for the resulting association strengths. And because these associations have a crucial role in planning actions in response to stimuli, subsequent stimulus–response translation is influenced by the strengths of these associations. As a result, action planning takes the contextual meaning (e.g., consistency among action effect features) of motor actions into account as represented in the acquired action–effect associations.

Importantly, Simulation 1 demonstrates that in HiTEC novel action effects become (at the distal level) associated to motor codes. The connection between sensory and feature code is the result of (earlier) grounding processes; the connection between motor code and feature code results from action–effect learning as demonstrated in both simulations. That is, execution of a motor code results in changes in the environment that are ‘picked up’ by sensory codes and distally represented by feature codes. Multiple encounters of this sensorimotor co-occurrence is considered to strengthen the connection between motor codes and feature codes. Moreover, different sensory codes may activate the same feature code. This is the case in Simulation 2 where both auditory and haptic sensory codes project to the same distal feature codes that code for ‘intensity’. Consistent action effects activate these feature codes more strongly and, therefore, lead to stronger action–effect associations as compared to inconsistent action effects, as suggested by the findings of Kunde and colleagues addressed in Simulation 2. Crucially, in the HiTEC simulation of this experiment, the auditory and haptic sensory codes receive equal external stimulation upon perceiving the action effect. It is due to the top down modulation of the task-feature connections with the ‘key’ code that the haptic sensations are enhanced and thus play a dominant role in representing the action effect as compared to the auditory sensations. It is this task set modulation of action effect representation (and its influence on action–effect learning and subsequent stimulus–response translation) that creates *situation*-*specific meanings of actions.*

Note that this flexibility is only possible because of the distinction that HiTEC makes between motor codes (which are unaffected by task set) and action–effect codes (which are affected by task set). To appreciate this point, consider the study by Wallace (1971). In one of his conditions, the participant’s left and right hands were held in parallel (i.e., placed on the spatially corresponding left and right keys) but they were crossed in another condition. In both conditions, left stimuli facilitated responses with the left key and right stimuli facilitated responses with the right key, irrespective of which hand was used to operate the key. In other words, what mattered for stimulus–response compatibility was the spatial relationship between stimuli and hand locations, but not the relationship between stimuli and particular hands. Considering that the motor program driving the keypress response of a particular hand is largely the same whether the hand is located on the left or right side of one’s body, it is impossible to model such a finding with a model that connects stimulus to motor codes—as the compatibility model suggested by Zorzi and Umilta (1995) and Kornblum, et al. (1990, 1999). Rather, stimulus codes must be related to some abstract action code that can be flexibly related to particular motor outputs (Wallace, 1971), exactly as suggested by HiTEC. It is this action or action–effect code, rather than motor codes, that intentional agents use to control their intentional actions—by “anticipating the intended action effects”, as ideomotor theory suggests. Given that they anticipate only the intended parts of all the action effects that a given action has been learned to produce, ideomotor action control can be considered to be a process comprising of attentional selection. If, for instance, a particular motor program (like pressing a key) is carried out by one’s right-hand located on the left side, the corresponding motor code is associated with both a “Left” code representing the hand’s location and a “Right” code representing its anatomical identity. What matters in the task like that used by Wallace (1971) is the location, agents would “attend” and “intentionally weigh” the “Left” code more/higher than the “Right” code, which effectively renders the action “Left”—a situation-specific meaning.

This first version of HiTEC considers action–effect learning as a rather reactive/passive process. Interestingly, the basic principle of picking up sensorimotor (or action-perception) contingencies is consistent with the sensorimotor theory of O’Regan and Noë (2001). In their approach, perceiving is a way of acting, actively exploring the environment rather than merely registering and representing the outside world, so that the process of active seeing (which includes the movement of body, head, and eyes) is directly producing the very changes that are picked up by visual receptors. The active perceiver/actor, so O’Regan and Noë argue, would need to learn how his/her own actions influence perceptions (sensorimotor contingencies) and perceiving the world builds on this knowledge. Although HiTEC does not model learning such contingencies per se, it does share the idea of acquiring grounded representations of sensorimotor regularities in interactions with the world and using those representations both for perception (as suggested in the sensorimotor theory) and actions, which indeed lead to perception, both in the sensorimotor account and in HiTEC.

One possibility to endow HiTEC with the more active learning strategy that O’Regan and Noë suggest is by means of action monitoring. The anticipated action effects are a trigger for action selection, but also form an expectation of the perceptual outcome of the action. Differences between this expectation and reality lead to adjusting the action on a lower sensorimotor level than is currently modeled in HiTEC. What matters now, is that the feature codes are interacting with the sensory codes, making sure that the generated perception is within the set parameters, as determined by the expected action outcome. If this is not (well enough) the case, the action should be adjusted. However, when a discrepancy of this expectation drastically exceeds ‘adjustment thresholds’, it may actually trigger action effect learning (phase 1). Apparently, the action–effect associations were unable to deliver an apt expectation of the actual outcome. Thus, anticipating the desired outcome falsely led to the execution of this action. This may trigger the system to modify these associations, so that the motor codes become associated with the correct action effect features. Such a monitoring system could work along the lines of (Botvinick, Braver, Barch, Carter, & Cohen, 2001). The experience of response conflict and/or of negative feedback might strengthen the activation state of goal codes and their impact on stimulus–response processing, which would tend to prevent errors in the future (van Steenbergen, Band, & Hommel, 2009).

Furthermore, anticipation based action control (see also Butz & Pezzulo, 2008; Haazebroek & Hommel, 2009) is consistent with basic concepts in research on human motor control (e.g., Wolpert & Ghahramani, 2000). Here, the motor system is considered to form a loop in which motor commands lead to muscle contractions which cause sensory feedback, which in turn influences future motor commands. Neural circuits are considered to form internal models that control motor action: forward models are considered to model the causal relationship between actions and their consequences and inverse models determine the motor command required to achieve the desired outcome. These concepts clearly resonate with the role of action–effect associations in the HiTEC model. Also, our approach is much in line with the work by Herbort and Butz (2012) who used a computational model to test action–effect learning in a similar way. Their model consists of action codes and effect codes only, and their simulations focused on various conditions of actions and effects (e.g., number of actions and effects, noise in effect representations, actions executed in parallel, different actions with the same effects, sequences of actions et cetera). In contrast, HiTEC also addresses stimulus perception and how stimulus perception and action planning using common codes may result in compatibility effects.

### Automaticity

Phenomena of stimulus–response compatibility are commonly considered to reflect the parallel processing of task-relevant information (such as color in a Stroop task) and task-irrelevant information (such as word meaning in a Stroop task). Accordingly, models of such phenomena distinguish between an intentional route, which processes instructed, task-relevant information, and an automatic route, which processes irrelevant information (e.g., Cohen, et al., 1990; Kornblum, et al., 1990, 1999; Zorzi & Umilta, 1995). The degree to which automatic processing occurs is assumed to depend on the strength of associations between stimulus and response codes, which either is assumed as a not further explained given (Kornblum, et al., 1990, 1999; Zorzi & Umilta, 1995) or attributed to stimulus–response learning.

HiTEC can also be construed as a dual-route model that processes relevant and irrelevant information in parallel, and the degree to which that occurs also depends on learning. And yet, the logic underlying the processing of irrelevant information is different and the degree to which that occurs is entirely independent of stimulus–response learning. First, “automatic” processing of actually task-irrelevant information occurs if and because there is feature overlap between stimulus and response representations. Although some other models have considered feature overlap as an important criterion (Kornblum, et al., 1990, 1999), they have failed to explain *how* this overlap translates into automatic processing—they simply assume that it does.

HiTEC provides a straightforward theoretical reason: if the stimulus event feature-overlaps with an action event, their representations are partially identical, which means that activating one necessarily activates the other to some degree. Second, the intentional-weighting principle of TEC and HiTEC implies that feature overlap can render task-irrelevant feature dimensions task-relevant. Take the Simon task: the emphasis of task instructions on response location will induce the intentional weighting of the location dimension. Given that HiTEC does not distinguish between stimulus location and response location, this means that increasing the weight of the response-location dimension necessarily implies higher weights on stimulus location. Accordingly, HiTEC considers automaticity a byproduct of intentional task preparation (Hommel, 2000). Note that this automaticity does not rely on stimulus–response learning (and can thus occur for entirely novel stimulus–response combinations) but on action–effect learning, through which actions acquire their features.

This logic is illustrated in Simulation 3, where stimulus–response compatibility/automaticity follows from the fact that responses are coded in terms of their spatial perceptual consequences, due to ideomotor learning. In similar vein, in Simulation 4, the task-irrelevant word feature only has influence because the response is coded in terms of these features (a result from action–effect learning). If the response would not be verbally defined (e.g., but in terms of key presses, which in HiTEC would be spatially represented) the compatibility effect is indeed dramatically reduced or eliminated, especially in tasks preventing internal naming (MacLeod, 1991).

### Task demands

In line with TECs general ‘intentional weighting’ principle (Memelink & Hommel, 2013), HiTEC explicitly addresses how task instructions are implemented in terms of representations and connections, and how they affect subsequent processing. The model is initially as task ignorant as humans are, until it ‘receives the task instruction’. A task instruction is implemented by connecting feature codes and task codes following the actual task rules in terms of stimulus features and response (i.e., action effect) features. Here, we hypothesize that feature codes can be accessed by means of verbal labels and that receiving a task instruction can activate these feature codes and connect them to generic task codes (i.e., some sort of internal simulation of the translation from stimulus features to response features). This allows the task instruction to be readily internalized as connections from feature codes to task codes to feature codes. As an example, the instruction “when you hear a high tone, press the left key” (taken from Simulation 5) would be implemented as connections from ‘High’ to ‘T_1_’ and from ‘T_1_’ to ‘Left’ and ‘Key’. These connections subsequently enable the model to produce stimulus–response translations in accordance with task demands. Note that apart from this instruction-based wiring we do not assume any other type of task-specific addition to the model (i.e., no additional ‘task inputs’ or biases during stimulus–response translation as is the case in the model of Cohen, et al., 1990).

These feature code–task code connections have two main consequences for subsequent processing: (1) they propagate activation from stimulus features towards response features that in turn excite motor codes and (2) they top down modulate lower level processing due to their recurrent nature. Arguably, this essentially constitutes ‘attention’: sensory codes that are connected to feature codes are enhanced, sensory codes that are not connected (i.e., stimulated by ‘the other’ elements in the scene) are not enhanced. Moreover, relative higher activation of feature codes also results in relative stronger enhancement of sensory codes. This enhancement of lower level representations is crucial both in stimulus–response translation (i.e., responding to a stimulus is an integrated process) and during action–effect learning where it focuses attention on the relevant action effect features.

Moreover, as stimuli and responses are both defined in terms of common feature codes, it could happen that a response feature code included in the task set may be activated by a sensory code due to perceiving a (albeit task-irrelevant) stimulus feature (automatic task cuing). In that case the feature code would receive both exogenous excitation directly originating from a sensory code due to stimulus perception and endogenous excitation originating from response planning. Note that the latter form of excitation indirectly also originates from the stimulus but is mediated by the task set. As a result, response planning may be facilitated or hampered due to interaction between these pathways, yielding stimulus–response compatibility (SRC) effects.

Crucially, these effects depend on both stimulus and response coding. In HiTEC, responses are coded in terms of their perceptual effects. During the action–effect learning phase, associations are strengthened between motor codes and feature codes. The strengths of these associations depend on the co-activation of these motor codes and feature codes, which depends on both external stimulation (not explicitly modeled in current simulations, all sensory codes receive the same external input when excited) and top down modulation (i.e., connectivity between sensory codes, feature codes and task codes). Thus, as stated above, the task set not only determines actual stimulus–response translation (both controlled and automatic), it also influences how responses are coded (during action–effect learning) and thereby how (controlled and automatic) subsequent stimulus–response translation is carried out.

This influence of task instruction on stimulus–response translation is explicitly demonstrated in Simulation 5, where the instruction based connections automatically result in specific recurrency that selectively enhances either the ‘key’ or ‘light’ feature codes that in turn selectively enhance either the haptic location codes or the visual location codes when perceiving action effects. This leads to differences in action–effect weight learning and subsequently in how a response is encoded. These differences in response coding, in turn, influence the degree in which the feature codes representing stimuli and responses overlap, giving rise to different SRC effect directions across conditions.

Note that all simulated studies are based on the main assumption that in experimental settings human participants generally only respond to particular stimuli with particular responses because they are instructed to do so. Indeed, it has been shown that the stimulus-induced response activation that underlies SRC effects can only be obtained after the participant has implemented the required task set (Valle-Inclán & Redondo, 1998). In this study, the stimulus–response mappings of the task (i.e., the task instruction) varied randomly from trial to trial. In some trials, the mapping instruction was presented followed by the target stimulus. In other trials, the stimulus preceded the mapping instruction. Their results showed that the Simon effect was only observed in trials where the mapping instruction was presented *before* the stimulus. This suggests the task set must be implemented before SRC can occur.

Thus, understanding the task demands configures the cognitive system to modulate both stimulus perception and response planning. This involves attending to task-relevant stimulus features (e.g., a high or low auditory pitch) and preparing a small selection of motor schemas (e.g., pressing keys). In more general terms this process of configuring the cognitive system is what we consider the main contribution of cognitive control; it prepares the system to subsequently act according to instruction—it in a sense turns the system into a ‘prepared reflex’ (Hommel, 2000; see also Bargh, 1989). Note that instruction wiring by itself ensures attention for the right dimension(s), for example key locations vs. light locations in Simulation 5. Indeed, as cognitive control is implemented as mere connectivity resulting from task instructions, there is—at least with respect to the simulated experiments—no need for additional online control of the inner mechanisms.

Addressing the apparent crucial role of task goals in SRC, Ansorge and Wühr (2004) formulated the *response*-*discrimination hypothesis* that states that response representations are not automatically formed, but rather top-down controlled. Only spatial features that discriminate between alternative responses are represented and thus give rise to a Simon effect. This resonates well with the conclusions in a general review by Proctor and Vu (2006) that the Simon effect does not result from an automatic activation of a corresponding response by means of a hard-wired (e.g., Kornblum, et al., 1990) or over-learned (e.g., Umiltà & Zorzi, 1997) route; rather the task defines S–R associations that mediate this responding. HiTEC is clearly consistent with this response-discrimination hypothesis and provides a rationale, in terms of internalization of an explicit task set using codes that are grounded in sensorimotor experience, for *how* and *why* these response representations are formed and top down modulated.

### Limitations and future work

Although we have demonstrated that the current version of HiTEC can simulate a variety of perception–action experiments, its principles, processes, and properties are still far from sufficient to account for all known phenomena in this domain. Most notably, HiTEC yet lacks the ability to bind features—an ability that was emphasized in the original TEC (Hommel, et al., 2001). When an agent is presented with two visual objects, say one blue and one red, both ‘Blue’ and ‘Red’ sensory codes will be activated concurrently and the present model has no means to code or keep track which color belongs to which object (the classic ‘binding problem’: Treisman, 1996). Given that several empirical phenomena in the domain of perception–action interactions are likely to reflect feature-binding processes (Müsseler & Hommel, 1997; see Hommel, 2004, for an overview), extending HiTEC to include a binding mechanism seems essential. From a modeling perspective, such a binding mechanism may protect active representations from catastrophic interference (see Hommel, et al., 2001). Closely related is the impact of episodic memory on perception and action planning (Waszak, Hommel, & Allport, 2003). Not only the current task set but also recent experiences with particular stimuli and actions (e.g., affective connotations, Haazebroek, van Dantzig, & Hommel, 2009) under a particular task set can play a large role in the later interpretation of stimuli and responses and the efficiency with which their processing can be controlled (Nuxoll & Laird, 2004).

In addition, action planning in HiTEC is currently greatly simplified as we only allow one rather simple action to be planned at a time. A more realistic model of action control would include the planning, programming, and coordination of more complex, hierarchical actions (Logan & Crump, 2010) and action sequences (Herbert & Butz, 2012; Tubau, Hommel, & López-Moliner, 2007). Moreover, the notion of planning may imply intention, a deliberate action actively chosen by an actor, rather than a response. Indeed, we have not addressed intention explicitly and have focused primarily on responding to stimuli. However, it is conceivable that also intended action may be realized by actively anticipating action effects (see Hommel, 2009 for a more detailed discussion). Another strong addition would be the inclusion of conflict monitoring and performance feedback along the lines of (Botvinick, et al., 2001). The experience of response conflict and/or of negative feedback might strengthen the activation state of goal codes and their impact on stimulus–response processing, which would tend to prevent errors in the future (van Steenbergen, et al., 2009).

Nevertheless we hope to have shed some light on the link between perception and action codes in cognitive processing across different experimental paradigms. Although HiTEC may seem to be a simple model considering its architecture, its recurrent connections yield complex dynamics. HiTEC complements available models by providing a rationale for *why* some stimulus–response relationships are privileged, and how they can be both automatic and context-dependent at the same time, how situation-specific meanings of responses emerge, and how they modulate sensorimotor processing. The HiTEC model is intended as a proof of principle, how common representations may mediate the interaction between perception and action in a stage-less way, addressing issues of action knowledge, automaticity and task demands, all relevant for effective behavior.

## Appendix

See Table 1.

**Table 1:** Parameters and values as used in the HiTEC simulations discussed in this article

Category	Parameter	Value
External inputs	External input (stimulus, motor execution)	0.5
Sensory weights	Sensory—feature forward	0.4
	Sensory—feature backward	3.0
Task—feature weights	(Stimulus) feature—task forward, and task—(response) feature forward	1.3^a^
	(Response) feature—task backward, and task—(stimulus) feature backward	0.2
Inhibition	Excitatory to inhibitory paired unit	1.25
	Inhibitory to other excitatory codes within layer	−0.75
Activation parameters	*d*_a_ Decay parameter	0.1^b^
	qa Sigmoid parameter in response function	0.9
	na Sigmoid parameter in response function	4
	*γ* Scale parameter	0.9
Noise	Mean	0.025
	Standard deviation	0.001
Thresholds	Voltage threshold (VT)	0.5
	Learning threshold (LT)	0.55
	Response threshold for motor code selection	0.7
Weight parameters	Weight decay (*d*_w_)	0.0005

^a^0.8 for ‘other’ features (i.e., key, light, sound)

^b^0.2 for sensory codes

## Footnotes

1 In HiTEC, action-effect learning internalizes the regularities in co-occurrences of motor actions with their perceptual effects. In our view this is most simply captured by a Hebb-like unsupervised learning rule based on co-activation, in similar vein as in the models by Zorzi and Umilta (1995) and Herbort and Butz (2012).

2 For the sake of simplicity, these feature codes are taken to represent all object characteristics that are not represented by other, specific feature codes, such as its color or location.

3
*t*(38) = 53.42, *p* < 0.001.

4
*t*(38) = 39.00, *p* < 0.001. Since we opted for fixed parameters and weights (when not learned) across instances, the model’s dynamics are slightly different due to the varying number of codes and connections between the instances. In Simulation 2 this results in a rather small effect size.

5
*F*(2,56) = 11,230.03, *p* < 0.001.

6
*F*(2,56) = 11,230.03, *p* < 0.001.

7 Analysis of variance shows a significant interaction effect between ‘Key’ vs ‘Light’ and stimulus-key congruency on response time (*F*(2,114) = 489.17, *p* < 0.001). Specific analysis of Key model trials shows a significant main effect for stimulus-key congruency (*F*(2,57) = 165.93, *p* < 0.001). Also, the specific analysis of Light model trials shows a significant main effect for stimulus-key congruency (*F*(2,57) = 767.62, *p* < 0.001).
